# Supplementary material for: Exploiting endogenous fibrocartilage stem cells to regenerate cartilage and repair joint injury
Source: Nat Commun. 2016 Oct 10;7:13073. doi: 10.1038/ncomms13073 (PMC5062541; doi:10.1038/ncomms13073)
Supplement: Supplementary Information — Supplementary Figures 1-10 and Supplementary Tables 1-4 [file ncomms13073-s1.pdf]

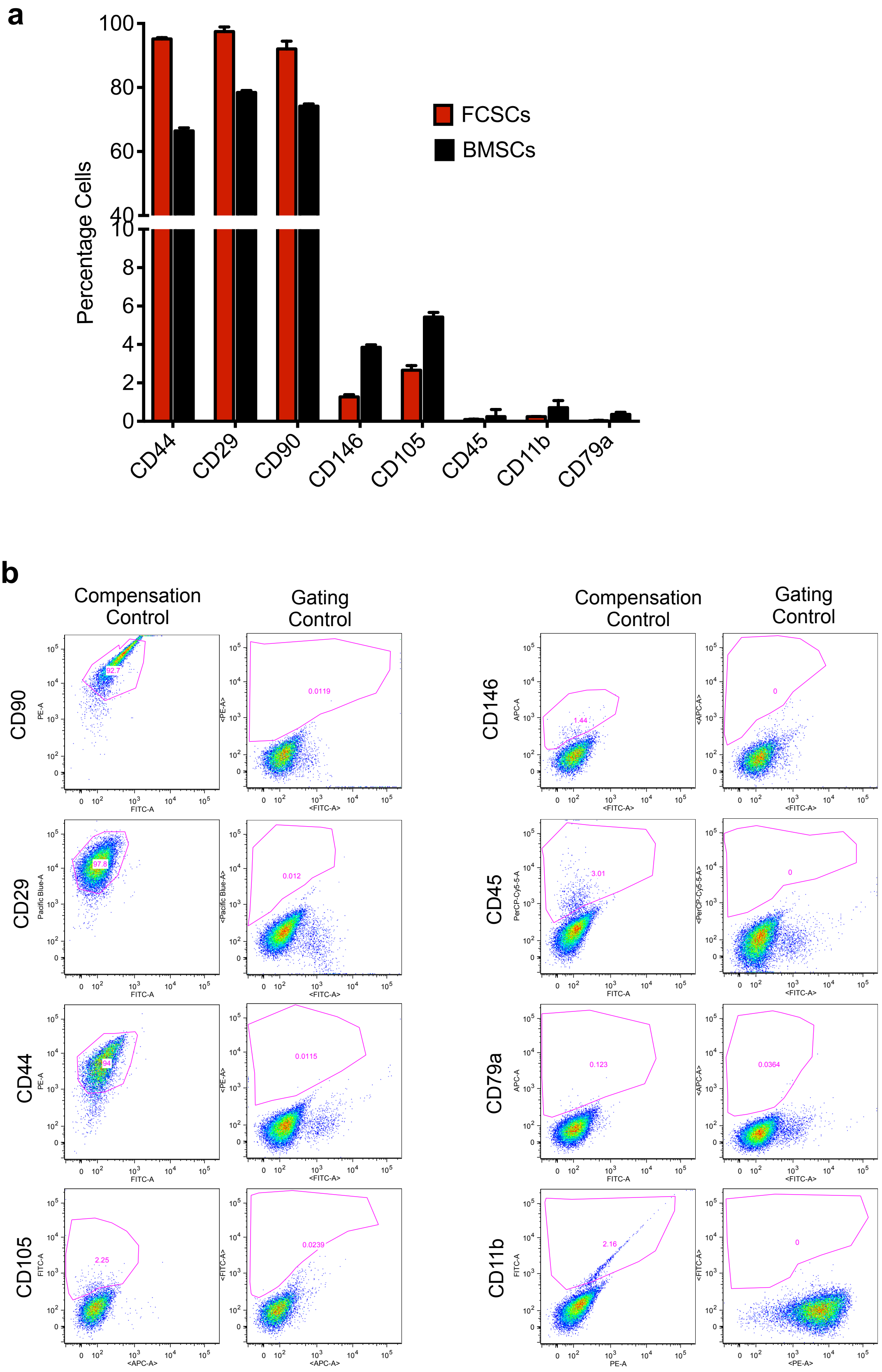

<u>Supplementary Figure 1:</u> FCSCs cell surface markers

(a) Flow cytometric analysis of FCSCs and donor-matched BMSCs. Data are mean ±S.D.; n=4 experiments. **(b)** Representative gating strategy for FCSCs. To determine positive cells compensation control including single fluorochrome and gating control excluding single fluorochrome were used.

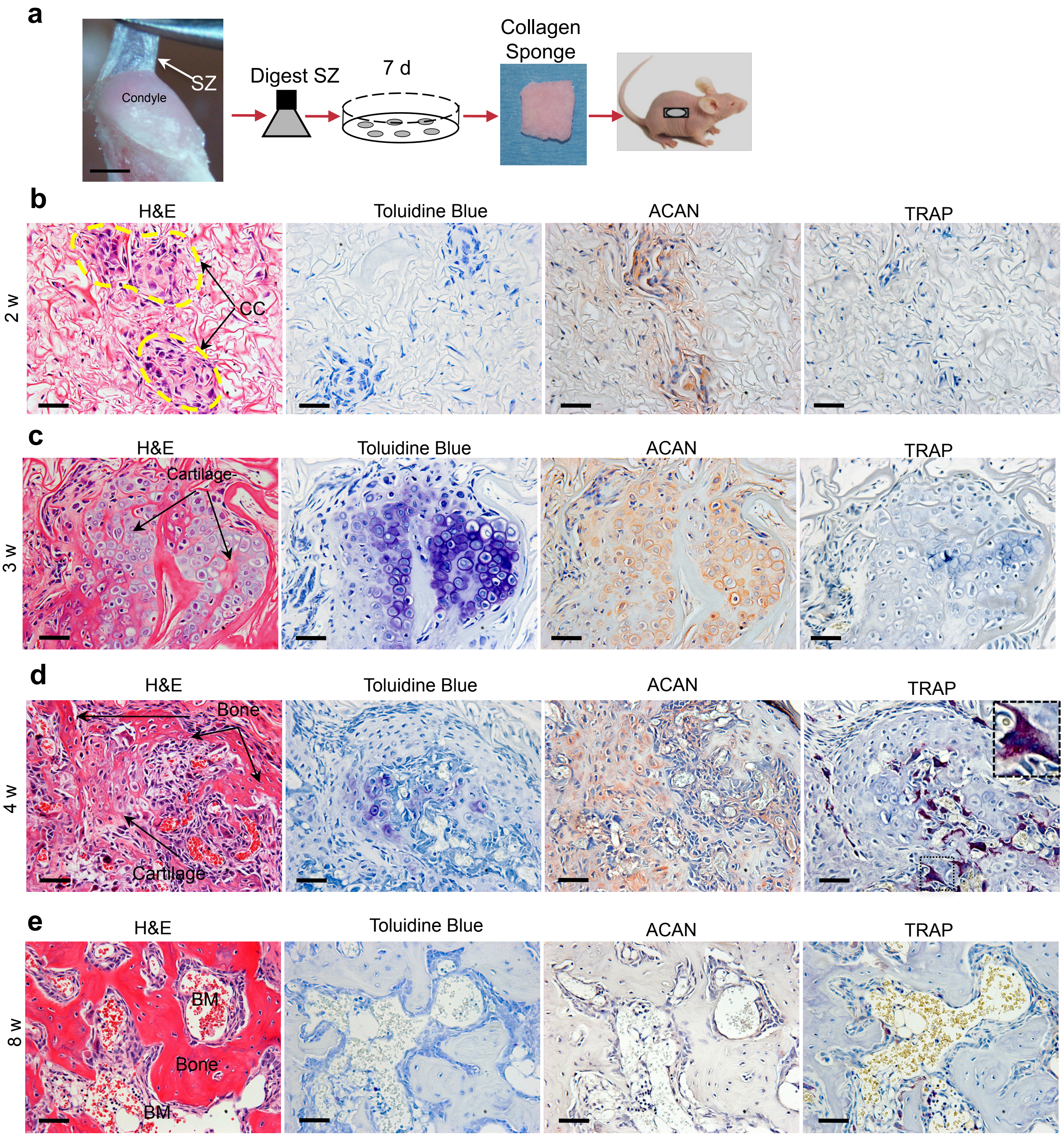

Supplementary Figure 2: FCSCs recapitulate endochondral ossification *in vivo*
(a) Schematic demonstrating FCSC transplantation experiment. Scale bar = 2mm. (**b-e**) Time course of representative histological sections of regenerated tissue after 2 (4/6 transplants), 3 (5/6 transplants), 4 (5/6 transplants) and 8 (7/8 transplants) weeks in vivo. Serial sections showing hemotoxylin and eosin (H&E) stain, toluidine blue stain, aggrecan immunohistochemistry (ACAN) and tartrate-resistant acid phosphatase (TRAP) staining. BM=bone marrow. CC=cell condensation. Sections are representative images. Scale bar= 50 μm.

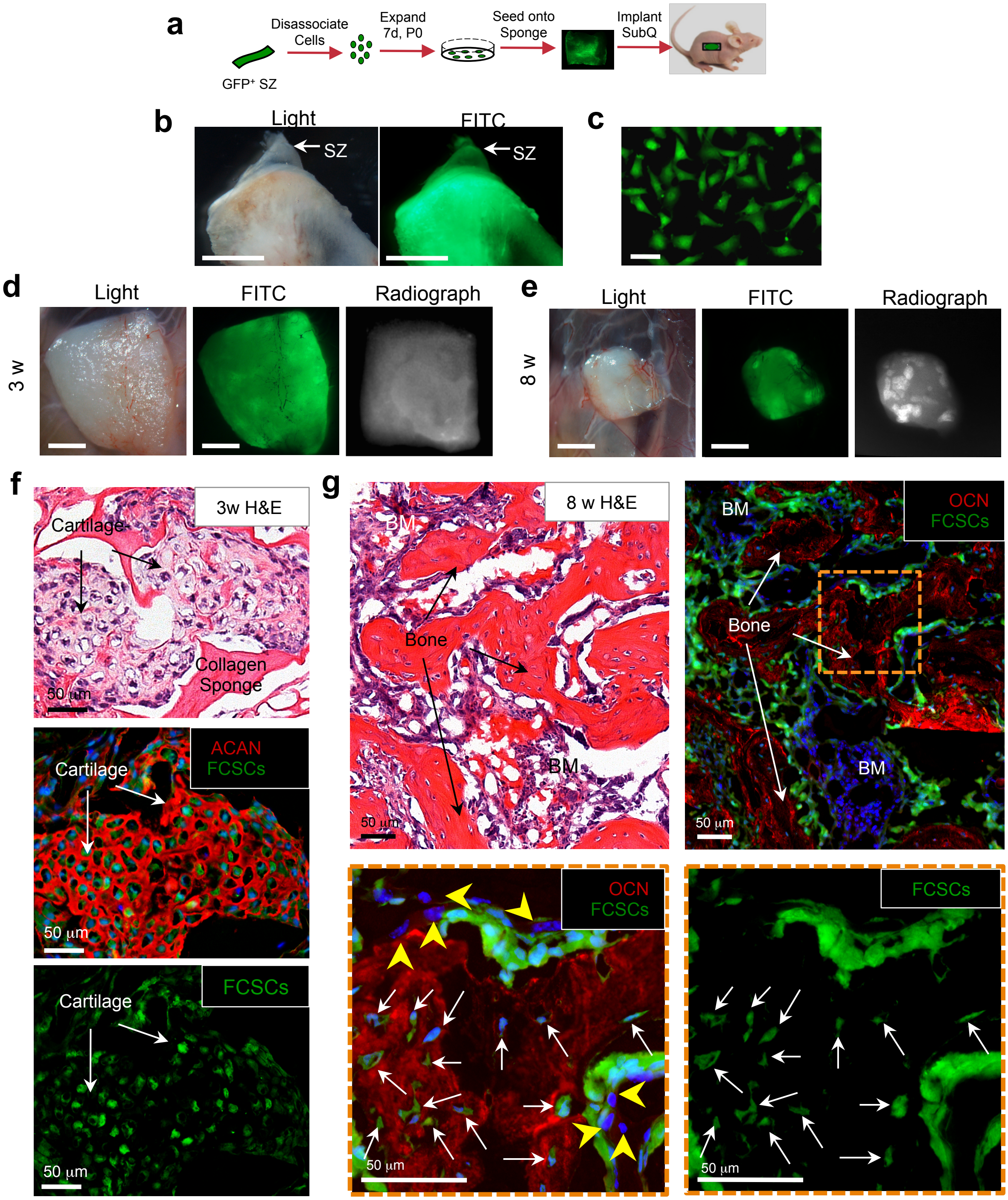

Supplementary Figure 3: GFP⁺ FCSCs spontaneously form cartilage and bone
(a) Schematic of GFP⁺ FCSC transplantation experiment. **(b)** GFP⁺ rat superficial zone tissue (SZ) dissection shown in light and fluorescent microscopy. Scale bar=4 mm. **(c)** GFP⁺ FCSCs shown at 5 days. Scale bar=50 µm. **(d,e)** GFP⁺ FCSC collagen sponge transplants on dorsum of nude mice after 3 and 8 weeks in vivo, respectively. Scale bar= 3mm. **(f)** Representative hematoxylin and eosin staining of GFP FCSC transplant after 3 weeks. Serial section showing GFP⁺ FCSCs under fluorescent light (green) and immunohistochemistry for aggrecan (acan, red). Dapi blue staining=nuclei. Scale bar=50 µm. **(g)** Representative hematoxylin and eosin staining of GFP FCSC transplant after 8 weeks. Serial section showing GFP⁺ FCSC under fluorescent light (green) and immunohistochemistry for osteocalcin (OCN, red). Dapi blue staining=nuclei. Scale bar=50 µm.

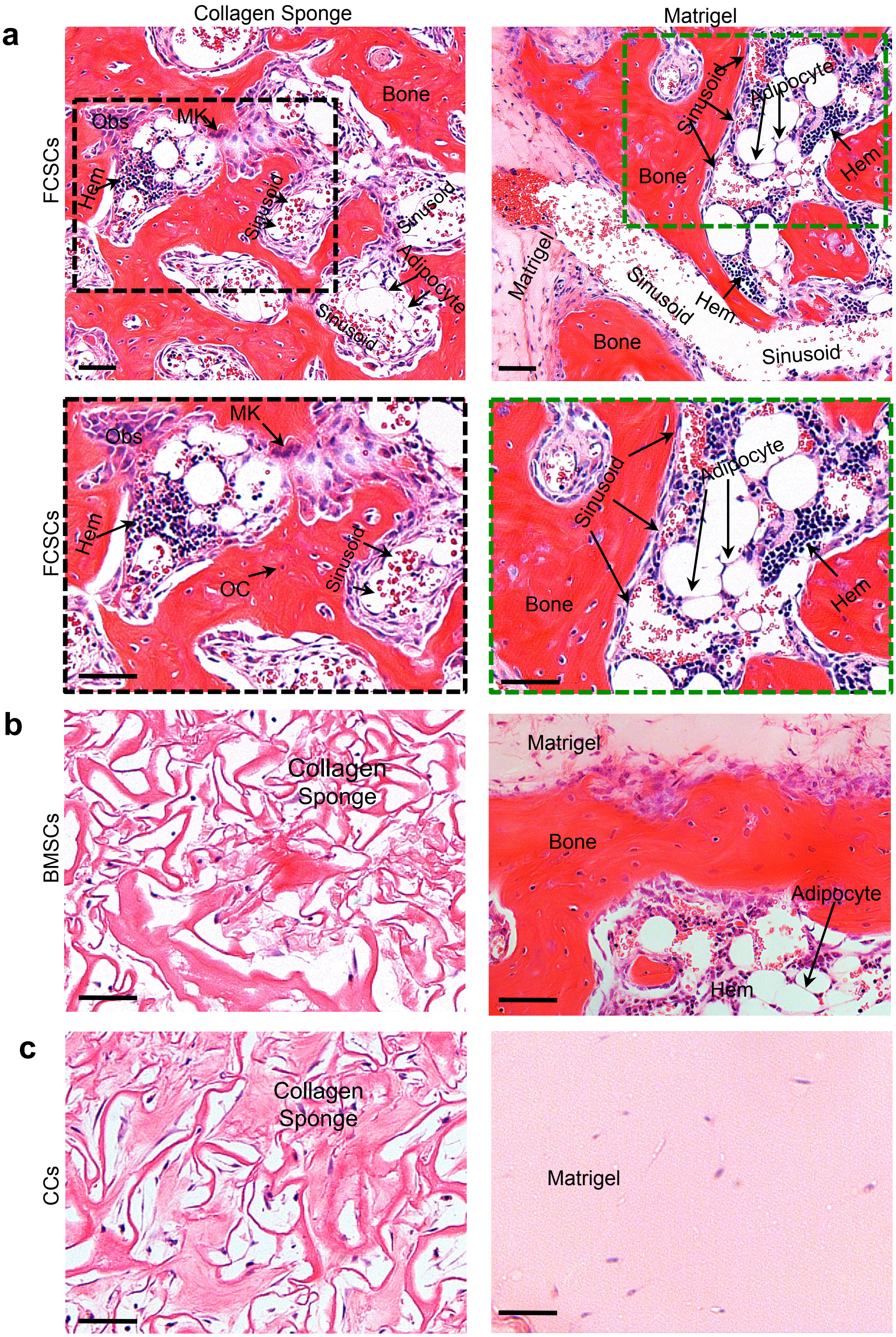

Supplementary Figure 4: FCSCs spontaneously generate organized bone
H&E staining of transplants show FCSCs generate bone with a hematopoietic microenvironment in multiple carriers after 8 weeks, but donor-matched condyle cells are unable to generate bone. Donor-matched FCSCs, bone marrow stromal cells (BMSCs), and condyle cells (CCs) were seeded onto **(a)** collagen sponge, **(b)** Matrigel or **(c)** gelfoam and transplanted on the dorsum on 8 week-old male nude mouse for 8 weeks. FCSCs form bone with mature osteocytes (OC) with an organized hematopoietic microenvironment including sinusoids, hematopoietic cell clusters (Hem), adipocytes, and osteoblasts (Obs). Scale bar = 50 μm.

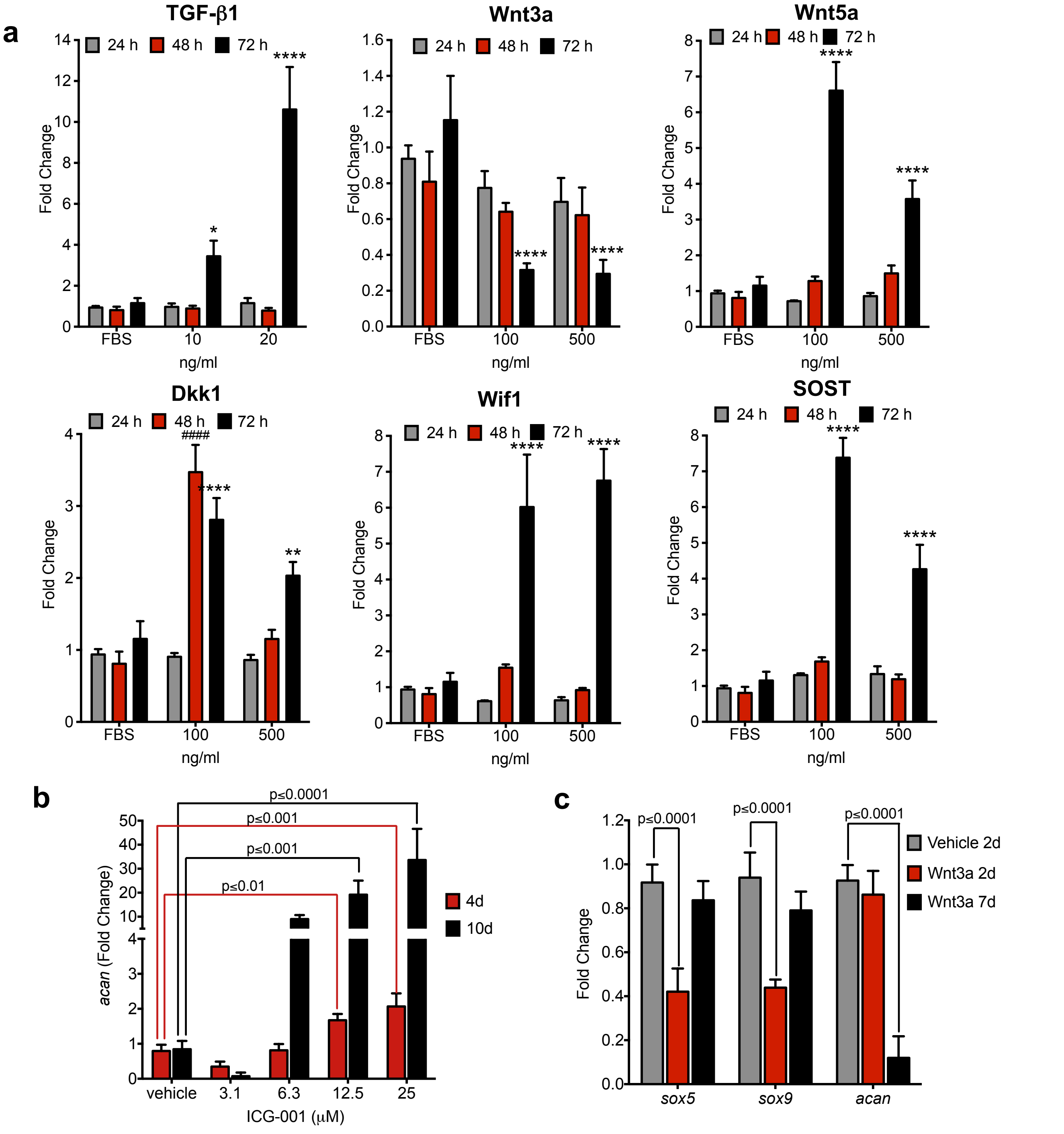

Supplementary Figure 5: FCSCs to differentiate into chondrocytes through inhibited Wnt
(**a**) FCSCs were treated with TGF-β1, Wnt3a, Wnt5a and canonical Wnt inhibitors (Dkk1, Wif1, SOST). qRT-PCR was used to determine aggrecan expression in FCSCs after 24h, 48h and 72h. Data are mean fold changed normalized to GAPDH and relative to FBS or vehicle control ± S.D.; n=3 independent experiments; two-way ANOVA followed by Tukey's post hoc test. (**b**) qRT-PCR of aggrecan (acan) expression in FCSCs with ICG-001 after 4d and 10d. Data are normalized to GAPDH and mean fold change relative to FBS/vehicle control ± SD; n=3 independent experiments; one-way ANOVA followed by Tukey's post hoc test. (**c**) qRT-PCR was used to *sox5*, *sox9* and *acan* expression in FCSCs treated with Wnt3a after 2d and 7d. Data are normalized to GAPDH and mean fold change relative to FBS/vehicle control 2 days ± S.D.; n=5 independent experiments; one-way ANOVA followed by Tukey's post hoc test.

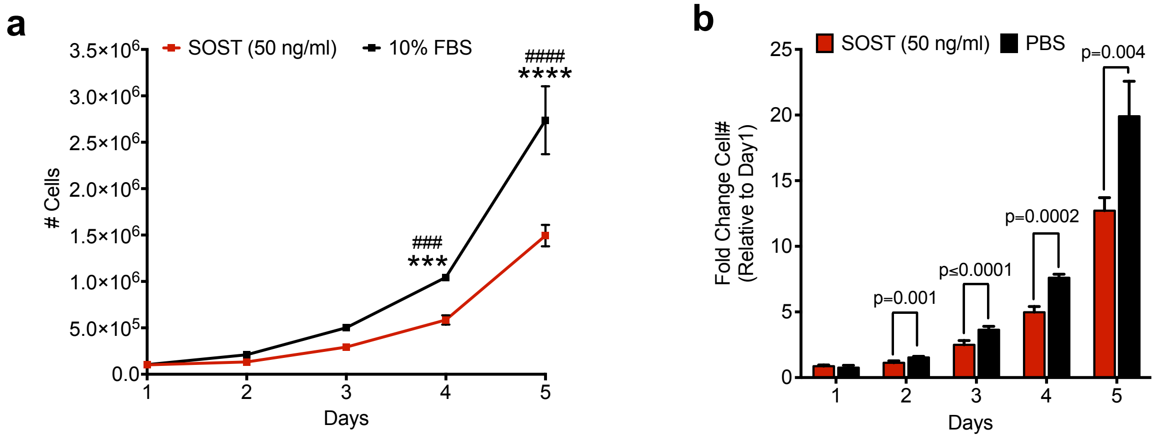

<u>Supplementary Figure 6:</u> Sclerostin inhibits FCSC proliferation

(a) Cell growth curve of FCSCs with SOST or vehicle over 5 days. Data are mean ± S.D.; n=6 independent experiments; ***p≤0.001 SOST 4d *vs* vehicle 4d; $^{###}$p≤0.001 SOST 1d *vs* SOST 4d; ****p≤0.0001 SOST 5d *vs* vehicle 5d; $^{####}$p≤0.0001 SOST 1d *vs* SOST 5d; two-way ANOVA followed by Tukey's post hoc test. (**b**) Data are mean fold change in cell number relative to 1d of SOST treated FCSCs and vehicle ± S.D.; paired Student's t-test.

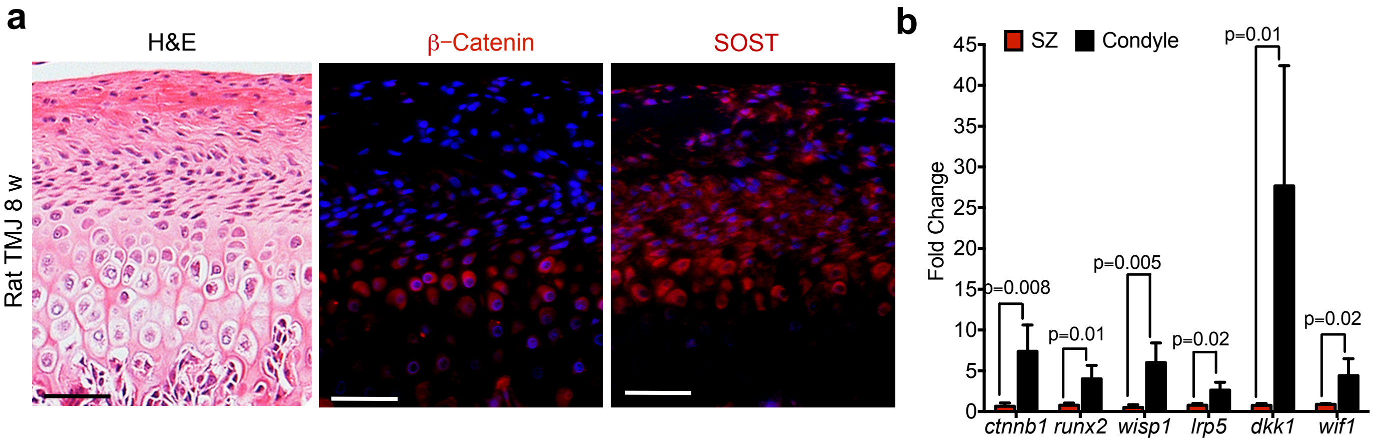

<u>Supplementary Figure 7:</u> Wnt signaling is temporally and spatially regulated in TMJ
(a) H&E and immunostaining for βCatenin (red) and sclerostin (SOST, red) in 8 week-old rat male TMJ condyle. Dapi=nucleus. Scale bar = 50 μm. **(b)** qRT-PCR for wnt target genes (*ctnnb1, runx2, wisp1, lrp5, dkk1*, and *wif1*) using RNA extracted from SZ (red) and condyle (black) tissues. Data are normalized to GAPDH and mean fold change relative relative to SZ tissue; error bars are S.D.; n=4 rats male rats 8w; paired Student's t-test.

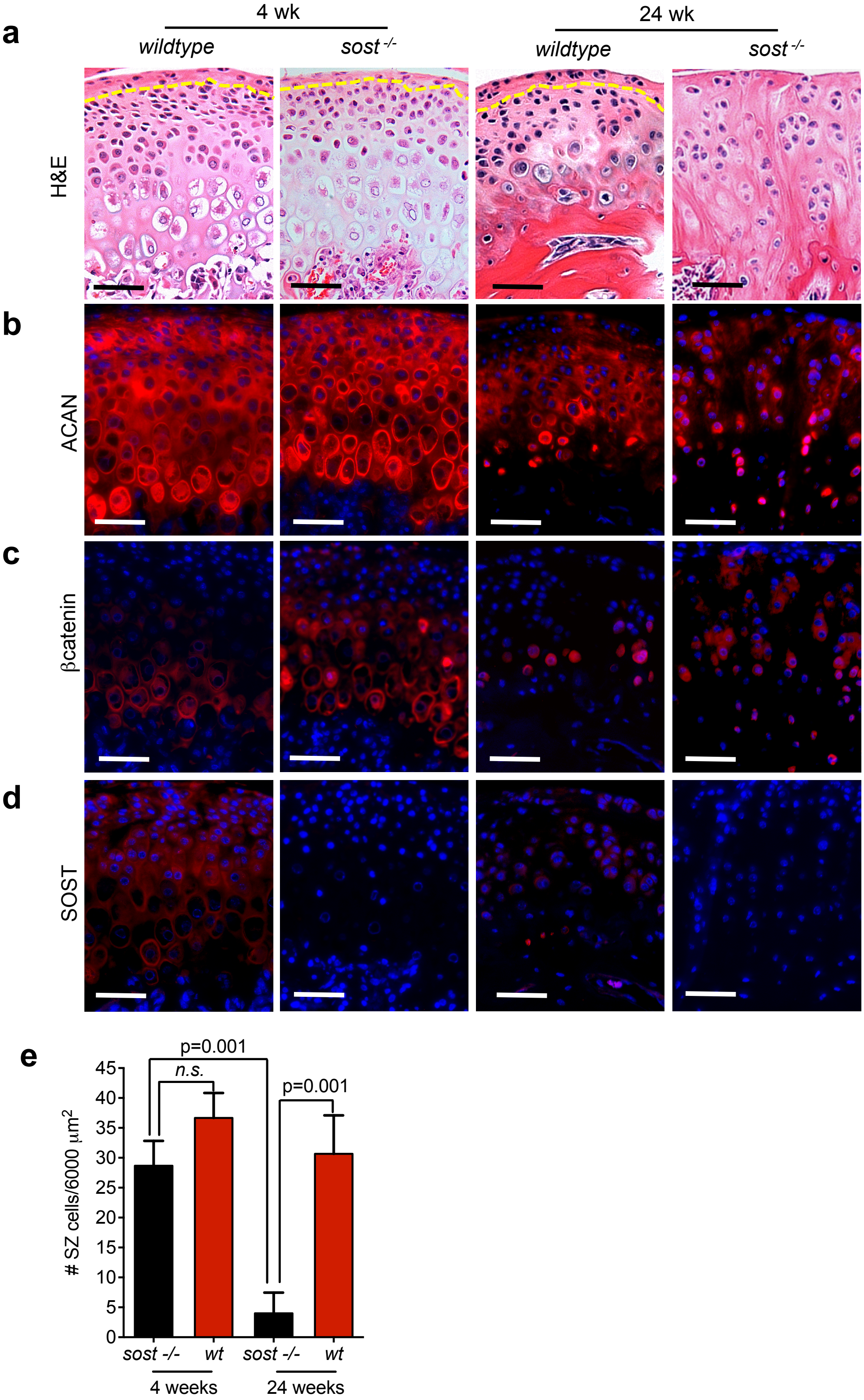

Supplementary Figure 8: Sclerostin deficiency in mice causes loss FCSCs
Mice deficient in sclerostin show decrease FCSCs, degeneration, and abnormal βcatenin and aggrecan distribution. Scale bar = 50 μm. **(a)** H&E staining and immunohistochemical staining of **(b)** aggrecan (ACAN), **(c)** βCatenin (βCat), and **(d)** sclerostin (SOST). **(e)** The number of FCSCs in SZ tissue in the comparable sagittal sections from WT and $sost^{-/-}$ mandibular condylar cartilages at 4 and 24 weeks of age. Data are mean; error bars are S.D.; n=3 $sost^{-/-}$ male mice 4 w and 24w; two-way ANOVA followed by Tukey's post hoc test.

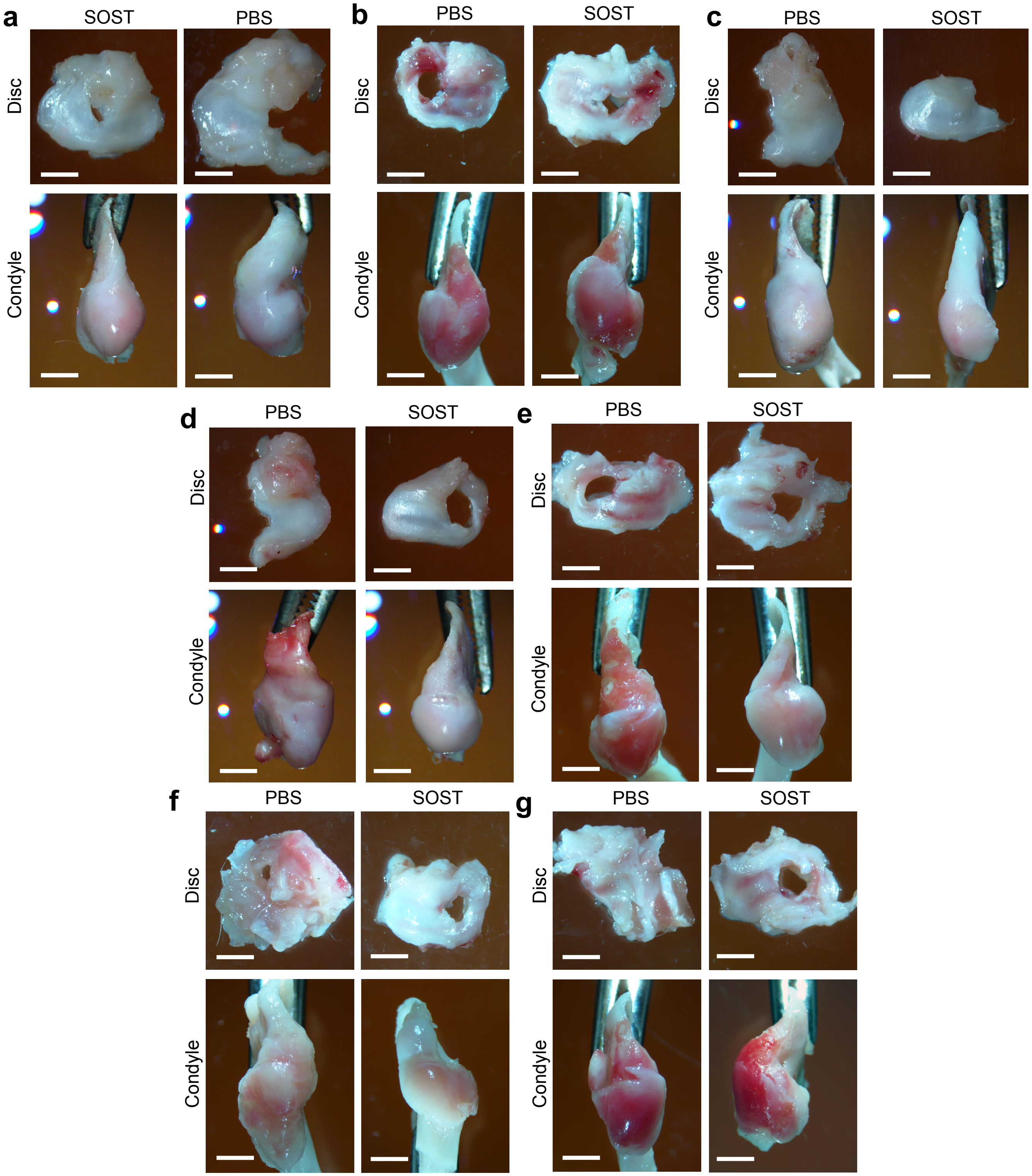

Supplementary Figure 9: Sclerostin ameliorates TMJ degeneration in rabbits.
(a-g) TMJ pathology was surgically induced in 12w-old male New Zealand white male rabbits and a punch biopsy was used to create a 2.5 mm perforation in the TMJ disc bilaterally. 3 days post-surgery, 0.1 ml SOST (100ng/ml in PBS) was injected into the inferior TMJ intra-articular space unilaterally on the right (**a**) or left (**b-g**) once weekly for 8 weeks. Vehicle control (PBS) was injected into the contra-lateral TMJ. Images are photographs that show the superior view of rabbit TMJ disc and condyle 8 weeks following bilateral disc perforation surgery. SOST was injected into the inferior TMJ intra-articular space unilaterally once weekly for 8 weeks, while vehicle control (PBS) was injected into the contra-lateral TMJ within the same animal. Scale bar = 3mm.

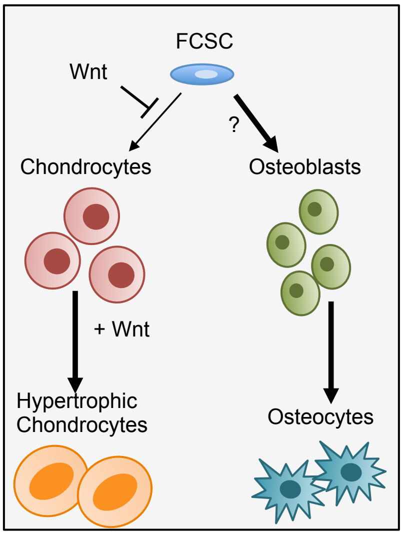

Supplementary Figure 10: FCSC fate specification is regulated by Wnt signaling.
A single FCSC can spontaneously commit to bone and cartilage lineages. Canonical Wnt signals inhibit FCSC differentiation into chondrocytes and promote chondrocyte terminal differentiation.

Supplementary Table 1: Single cell clonal multi-differentiation potential analysis
A total of 31 single cell FCSC clonal progenies were isolated and their ability to undergo adipogenesis, chondrogenesis and osteogenesis was tested *in vitro.*

Total Number of Clones Isolated	31 Clones
Tri-lineage Differentation Potential	22.5% (7/31 clones)
Bi-lineage Differentiation Potential	64.5% (20/31 clones)
Single Lineage Differentiation Potential	12.9% (4/31 clones)

Supplementary Table 2: Human TMJ fibrocartilage Analyses
12 patient samples were obtained from NIDCR TIRR that underwent TMJ condyle fibrocartilage replacement surgery · The osteoarthritic histopathological score was graded and β-catenin expression was quantified.

Research ID	Age DOS	Gender	Race	Stage	Grade	Score	# βcatenin cells/ μm^2 x 10^6
T06359	29	F	Asian	4	3.5	14	142.45
T06368	33	F	White	4	4.0	16	155.40
T06353	22	F	White	4	4.0	16	366.95
T08620	25	F	White	4	4.0	16	179.90
T06313	52	F	White	4	4.4	18	453.03
T06017	22	F	Unknown	4	4.5	18	449.35
T07616	35	F	White	4	4.5	18	540.35
T06554	41	F	White	4	5.0	20	351.44
T09634	39	M	White	4	5.0	20	366.55
T07610	52	F	White	4	6.0	24	334.79
T06309	38	M	White	4	6.0	24	178.19
T06347	42	F	White	4	6.0	24	100.65

Supplementary Table 3: Antibodies used for immunohistochemistry and flow cytometry

Primary Antibody	Source	Secondary Antibody	Isotype Negative Control
Rabbit anti-**Aggrecan**	Millipore AB1031 (1:100)	Invitrogen A-11010 (1:1000) or Invitrogen A-11008 (1:1000)	Rabbit IgG R&D AB-105-C
Rabbit anti-**Lubricin**	Novus Biologics 19048 (1:100)	Invitrogen A-11010 (1:1000)	Rabbit IgG R&D AB-105-C
Mouse anti-**Type II Collagen**	Millipore MAB8887 (1:50)	Invitrogen A-11003 (1:1000) or Invitrogen A-11001 (1:1000)	Mouse IgG1 R&D MAB002
Rabbit Anti-**Osteocalcin**	Millipore AB10911 (1:100)	Invitrogen A-11010 (1:1000)	Rabbit IgG R&D AB-105-C
Rabbit Anti-**β Catenin** (for mouse, rat, and human)	Abcam AB6302 (1:50)	Invitrogen A-11010 (1:1000, rodent) (1:2500, human)	Rabbit IgG R&D AB-105-C
Mouse Anti-**β Catenin** (for rabbit)	Millipore Mab2081 (1:100)	Invitrogen A-11001 (1:1000)	Mouse IgG1 R&D MAB002
Rabbit Anti-**Sclerostin**	Abcam AB63097 (1:50)	Invitrogen A-11010 (1:1000)	Rabbit IgG R&D AB-105-C
Mouse Anti-**CD90** (PE)	Abcam AB33694	N/A	Mouse IgG1 (PE) Abcam AB91357
Hamster Anti-**CD29 (**Pacific Blue)	Biolegend 102224	N/A	Hamster IgG (Pacific Blue) Biolegend 400925
Mouse anti-**CD44** (PE)	Abcam AB23396	N/A	Mouse IgG1 (PE) Abcam AB91357
Mouse anti-**CD105**	Novus Biologics NBP1-42383	Novus Biologics anti Mouse IgG Antibody (FITC) NB720-F	Mouse IgG1 R&D MAB002
Mouse anti-**CD146** (APC)	R&D FAB3250A	N/A	Mouse IgG2a (APC) R&D IC003A
Mouse anti-**CD45** (PerCp)	Biolegend 202220	N/A	Mouse IgG1 (PerCP) Biolegend 400150
Mouse anti-**CD79a** (APC)	Abcam AB188420	N/A	Mouse IgG1 (PE) Abcam AB67435
Mouse anti-**CD11b** (FITC)	BD Biosciences 554982	N/A	Mouse IgA (FITC) 553478

Supplementary Table 4: Primers used for qRT-PCR

Gene Symbol	Taqman Gene® Expression Assay
gapdh	Rn01775763_g1
sox6	Rn00488400_m1
sox9	Rn01751069_mH
acan	Rn00573424_m1
col2a1	Rn01637081_g1
col10a1	Rn01408030_m1
bgn	Rn00567229_m1
fmod	Rn00589918_m1
dcn	Rn01503161_m1
scx	Rn01504576_m1
col1a1	Rn01463848_m1
prg4	Rn01490812_m1
sox5	Rn01492418_m1
ppar-γ	Rn00440945_m1
ocn	Rn01455285_g1
twist1	Rn00585470_s1
ccnd1	Rn00432360_m1
ctnnb1	Rn00584431_g1
runx2	Rn01512298_m1
wisp1	Rn01505161_m1
lrp5	Rn01451428_m1
dkk1	Rn01501537_m1
wif1	Rn00586968_m1
